# Leaky Gut Driven by Dysbiosis Augments Activation and Accumulation of Liver Macrophages *via* RIP3 Signaling Pathway in Autoimmune Hepatitis

**DOI:** 10.3389/fimmu.2021.624360

**Published:** 2021-03-25

**Authors:** Hongxia Zhang, Man Liu, Weilong Zhong, Yanping Zheng, Yanni Li, Liping Guo, Yujie Zhang, Ying Ran, Jingwen Zhao, Lu Zhou, Bangmao Wang

**Affiliations:** ^1^Department of Gastroenterology and Hepatology, General Hospital, Tianjin Medical University, Tianjin, China; ^2^Department of Pathology, General Hospital, Tianjin Medical University, Tianjin, China; ^3^Department of Gastroenterology and Hepatology, People’s Hospital of Hetian District, Hetian, China

**Keywords:** autoimmune hepatitis, intestinal barrier, dysbiosis, macrophages, RIP3 signaling pathway, gut-liver axis

## Abstract

The gut–liver axis has been increasingly recognized as a major autoimmunity modulator. However, the implications of intestinal barrier in the pathogenesis of autoimmune hepatitis (AIH) remain elusive. Here, we investigated the functional role of gut barrier and intestinal microbiota for hepatic innate immune response in AIH patients and murine models. In this study, we found that AIH patients displayed increased intestinal permeability and pronounced RIP3 activation of liver macrophages. In mice models, intestinal barrier dysfunction increased intestinal bacterial translocation, thus amplifying the hepatic RIP3-mediated innate immune response. Furthermore, GSK872 dampened RIP3 activation and ameliorated the activation and accumulation of liver macrophages *in vitro* and *in vivo* experiments. Strikingly, broad-spectrum antibiotic ablation significantly alleviated RIP3 activation and liver injury, highlighting the causal role of intestinal microbiota for disease progression. Our results provided a potentially novel mechanism of immune tolerance breakage in the liver *via* the gut-liver axis. In addition, we also explored the therapeutic and research potentials of regulating the intestinal microbiota for the therapy of AIH.

## Introduction

Autoimmune hepatitis (AIH) is a chronic immune-mediated inflammatory liver disease. Although genetic and environmental factors are involved in the pathogenesis of AIH, the underlying mechanisms remain unclear (1). In recent years, great importance has been attached to the role of intestinal barrier in the pathogenesis of diverse immune-mediated diseases (2–4). In particular, the liver is continuously exposed to gut-derived antigens through the portal vein, which influence its innate and adaptive immune responses (5, 6). It’s known that intestinal barrier disruption can trigger bacteria and bacterial products translocation, which consecutively activate immune cells to release various proinflammatory cytokines and chemokines in the liver (7, 8). Clinically, primary sclerosing cholangitis is a remarkable example of chronic biliary inflammation highly associated with inflammatory bowel disease, indicating that the gut-liver axis plays an important role on the pathogenesis (9, 10).

Macrophages represent a key cellular component of the liver essential for maintaining tissue homeostasis and ensuring rapid responses to hepatic injury (11). Researches have reported the key role for liver macrophages in AIH. H Grønbaek et al. studied 121 AIH patients in a cross-sectional design and demonstrated macrophage activation paralleling disease activity, severity and treatment response, suggesting a role for macrophage activation in AIH (12). Besides, Assis David N reported a distinct genetic and immunopathogenic basis for AIH at the macrophage migration inhibitory factor locus, which indicated that macrophages play a role in pathogenesis and as biomarkers of AIH (13, 14). Liver macrophages consist of ontogenically distinct populations termed as Kupffer cells and monocyte-derived macrophages (15). As macrophages accumulate gut-derived products such as lipopolysaccharide (LPS) and undergone activation, necrosis of macrophages and uncontrolled release of inflammatory cytokine and chemokine results in inflammation and fibrosis of liver tissues (16, 17). Thus, macrophage cell death has been considered to be a major contributor of immune-mediated liver injury (18). Receptor interacting protein kinase 3 (RIP3) has been increasingly recognized as a central player in necroptosis and RIP3 kinase activity supports the recruitment of mixed lineage kinase domain-like (MLKL) to trigger membrane leakage with the consequent release of inflammatory cytokines and chemokines (19–21). Recently, we reported that probiotics application in experimental autoimmune hepatitis (EAH) mice could improve the intestinal barrier and downregulate the RIP3 signaling of liver macrophages (22). Hence, we hypothesized that the activation of RIP3 signaling pathway may be a potential mechanism of gut-liver axis in AIH pathogenesis and thus can be a novel treatment target.

In this study, we demonstrated that intestinal barrier damage and RIP3-mediated activation of liver macrophages existed in AIH patients. As reveled by the tandem model of dextran sulfate sodium (DSS) - concanavalin A (Con A), the disruption of intestinal barrier prior to hepatitis aggravated the activation and accumulation of liver macrophages. This finding highlighted RIP3 as an important interface that mediated liver inflammation. Furthermore, the RIP3-mediated activation of liver macrophage in EAH mice was canceled by gut sterilization, suggesting that immune responses in the liver are potentially regulated by gut microbiota.

## Materials and Methods

### Ethical Approval Statement

All experimental procedures were performed according to the guidelines of the Institutional Animal Care and Use Committee at Tianjin Medical University and followed the International Association of Veterinary Editors guidelines for the Care and Use of Laboratory Animal. The animal use protocol listed below has been reviewed and approved by the Animal Ethical and Welfare Committee of Tianjin Medical University, Approval No. IRB2020-WZ-119.

### Participants

Sixty-eight patients with AIH including thirty-nine without cirrhosis (AIH-n) and twenty-nine with cirrhosis (AIH-c) and fifteen controls were included. The patients were recruited from the Gastroenterology Department at Tianjin Medical University General Hospital. AIH was diagnosed with the following criteria: (1) patients conformed with 1999 revised International Autoimmune Hepatitis Group score≥10 and/or (2) 2008 IAIHG simplified AIH score≥6 and/or (3)histological features indicative of AIH (23, 24). Patients data were collected prior to corticosteroid therapy. The control subjects (CTRL) were selected from the Health Management Center of Tianjin Medical University General Hospital and matched the patients with AIH in terms of age and gender. Inclusion criteria for the CTRL group were as follows: (1) normal ranges of liver function test, (2) an absence of hepatitis B/C virus antigen, (3) normal abdominal ultrasound tests, and (4) an absence of autoimmune diseases and family history. Blood was collected from the individuals. Feces were collected from six AIH-n patients. Intestinal mucosal biopsy specimens were collected from fourteen patients (six AIH-n patients and eight AIH-c patients) and six controls. Liver biopsy specimens were collected from six AIH-n patients and four patients with hepatic cyst.

### Animal Experiments

Twenty-four female SPF C57BL/6 mice (6 weeks of age) were purchased from Beijing Animal Study Centre and reared under specific pathogen-free conditions in Animal Centre of the Tianjin Medical University. The mice were randomly divided into four groups (n=6 per group) including the CTRL group, DSS group, Con A group and DSS-Con A group. 1%DSS (MP Biomedicals) was dissolved in sterile distilled water ad libitum for 7 days to induce disruption of intestinal barrier integrity. Con A (15 mg/kg, Solarbio) was i.v. administered into the tail vein of mice 12 hours before liver resection (**Supplementary Figure 1A**). A RIP3 kinase inhibitor GSK872 (Merck) was diluted in 1 mg/mL dimethyl sulfoxide (DMSO). Another 12 C57BL/6 mice were randomized divided into two groups including DSS-Con A group and GSK872-pretreated group (n=6 per group) provided with 1% DSS water for 7 days and intraperitoneally treated with either GSK872 (1 mg/kg) or an equal volume of DMSO 1 h prior to Con A administration.

Another 26 female C57BL/6 mice were randomly divided into three groups (n=6 per group) including the CTRL group, EAH group and antibiotic mixture (Abx) group. The rest mice (n=8) were used to extract hepatic antigen S100 as previous description (25). All mice except the CTRL group were injected intraperitoneally with 0.5ml S100 emulsified in an equal volume of complete Freund’s adjuvant (CFA, sigma, USA) on day 7 and day 14 to induce EAH. The Abx group was pretreated with antibiotic mixture (0.5 g/L vancomycin, 1 g/L ampicillin, 1 g/L metronidazole, and 1 g/L neomycin; Sigma–Aldrich) for 2 weeks prior to S100 administration to deplete endogenous commensal microbiota. On day 28, all the animals were sacrificed under anesthesia (**Supplementary Figure 2A**).

### Fecal Supernatants Extraction

Fecal samples from patients with AIH were mixed at equal weight. One gram of the mixed feces was diluted in 5 mL sterile PBS solution, then initial filtered, concentrated, homogenized, step by step filtered, centrifuged. The supernatant was collected and centrifuged at 5,000 × g for 10 min at 4°C, then the supernatant of feces from AIH patients (AIH-s) was collected and filter-sterilized through 0.22 μm filters (26).

### Cell Line and Culture Conditions

Human Caco-2 cells (BNCC 338148) were cultured in Modified Eagle’s Medium (MEM) (Gibco) supplemented with 20% fetal bovine serum and a penicillin-streptomycin solution. The cells were incubated in a humidified incubator containing 5% CO_2_ at 37°C and were seeded in a 24-well plate at a density of 1×10^5^ cells per well. In the stimulation experiment (AIH-s group), the cells were pre-treated with 10% AIH-s for 24h, and the control groups were treated 10% inactive AIH-s or PBS for 24h. Mouse macrophage cell line RAW264.7 was plated in Dulbecco modified eagle medium (Gibco) supplemented with 10% FBS, 100 U/mL penicillin, and 0.1 mg/mL streptomycin (Gibco). The cells were cultured under same conditions as above. RAW264.7 cells were seeded in a 12-well plate at a density of 1×10^5^ cells per well. In LPS experiments (LPS group), the cells were treated with LPS (3 mg/mL, Solarbio Biotech) for 24 h. In GSK872 experiments (LPS-GSK872 group), the cells were treated with LPS (3 μg/mL) and GSK872 (3 µM) for 24 h.

### In Vivo Permeability Assay

Intestinal permeability was determined through fluorescein isothiocyanate (FITC)-dextran assay. FITC-D (4kDa, Sigma-Aldrich) was dissolved in normal saline infusion (50 mg/mL) and administered to mice through gavage at 6 mg/10 g body weight. Whole blood was collected 4h after FITC-D administration by using heparinized microhematocrit capillary tubes *via* eye bleed. Plasma was extracted from the blood through centrifugation at 4°C for 10 min at 3,000 rpm. Fluorescence intensity was analyzed using a plate reader. FITC-D concentration of each mouse was detected based on the FITC-D standard curve.

### Enzyme-linked Immunosorbent Assay (ELISA) and Biochemical Analysis

The blood was centrifuged at 3000 rpm for 10 min, and the plasma was then stored at −80°C. LPS, D-lactic acid (DLA), and diamine oxidase (DAO) plasma concentrations were quantified with ELISA kits (SenBeiJia Biotech) in accordance with the manufacturer’s instructions. Zonulin plasma concentrations were quantified using ELISA kits (Elabscience). Plasma alanine aminotransferase (ALT) and aspartate aminotransferase (AST) levels were tested by using the automated chemistry analyzer from the clinical laboratory of the Tianjin Medical University General Hospital.

### Histology and Immunohistochemistry (IHC)

The liver and intestinal tissues of patients and mice were collected and fixed in 4% paraformaldehyde. The paraffin-embedded liver and intestinal tissues were sectioned at approximately 5 µm and stained with hematoxylin and eosin (HE) following the standard HE protocol. Pathological changes in the liver and intestinal tissues were evaluated by two independent and experienced pathologists. Intestinal tissue sections from patients were stained with primary anti- zonula occludens-1 (ZO-1) antibody (ab96587, Abcam, Cambridge, MA, USA) or anti-Occludin antibody (ab216327, Abcam, Cambridge, MA, USA) at 4°C overnight and incubated with second antibody for 30 min at 37°C. The staining index was calculated by multiplying percentage positive cells rating by intensity rating in every field for quantitative analysis.

### Immunofluorescence

Caco-2 cell monolayers were fixed in cold methanol for 5 min at -20°C. Monolayers were then washed and blocked with bovine serum albumin (BSA) for 30 min at room temperature. Cells were then incubated with either anti-ZO-1 (1:50, Abcam, USA) or anti-Occludin (1:150, Abcam, USA) overnight at 4°C. Cells were then incubated with the secondary antibody for 1 h at room temperature. Cell nuclei were stained with DAPI. Double immunofluorescence analyses for liver macrophages were performed with 4 mm-thick frozen sections. Slides were fixed with acetone, blocked with 5% bovine serum albumin, and incubated with primary antibodies against CD68 (ab955, Abcam, Cambridge, MA, USA) and MAC387 (ab92507, Abcam, Cambridge, MA, USA) at 4°C overnight. The slices were restored to room temperature the next day, incubated with the corresponding secondary antibodies for 1h at 37°C, underwent DAPI reaction, sealed, and observed under a fluorescence microscope.

### Quantitative Real-Time PCR (qRT-PCR)

Total RNA was extracted from liver tissues with TRIzol (Thermo Scientific Inc.), followed by cDNA reverse transcription using the FastKing RT kit (TIANGEN). Real-time-PCR was performed using SYBR^®^ Select Master Mix (Thermo Scientific Inc.). Oligonucleotide primers for target genes are listed in **Table 1** and **Table 2**. Glyceraldehyde-3-phosphate dehydrogenase (GAPDH) was employed as an endogenous control. The relative mRNA expression levels of the target gene were evaluated by calculating the fold-changes normalized to the GAPDH for each sample using 2^−ΔΔCt^ methods. All cDNA samples were analyzed in triplicate.

**Table 1:** The Oligonucleotide primers used in Realtime-PCR analysis.

Human gene	Primer sequences (5^′^- 3^′^)
GAPDH	Forward primer: CCCTTCATTGACCTCAACTACATGG Reverse primer: CATGGTGGTGAAGACGCCAG
TNF-α	Forward primer: ACTCCAGGCGGTGCCTATG Reverse primer: GAGCGTGGTGGCCCCT
IL-6	Forward primer: CCAGTTGCCTTCTTGGGACT Reverse primer: GGTCTGTTGGGAGTGGTATCC
IL-1β	Forward primer: GTGGCTGTGGAGAAGCTGTG Reverse primer: GAAGGTCCACGGGAAAGACAC
CCL2	Forward primer: TTTTCCCCTAGCTTTCCC Reverse primer: GCAATTTCCCCAAGTCTCT
CCR2	Forward primer: AGGGCTGTATCACATCGG Reverse primer: ACTTGTCACCACCCCAAA
RIP3	Forward primer: TCCAGGGAGGTCAAGGC Reverse primer: ACAAGGAGCCGTTCTCCA
MLKL	Forward primer: TTCACCCATAAGCCAAGGAG Reverse primer: GGATCTCCTGCATGCATTTT

**Table 2:** The Oligonucleotide primers used in Realtime-PCR analysis.

Murine gene	Primer sequences (5′- 3′)
GAPDH	Forward primer: TGTGTCCGTCGTGGATCTGA Reverse primer: CCTGCTTCACCACCTTCTTGA
ZO-1	Forward primer: GGGCCATCTCAACTCCTGTA Reverse primer: AGAAGGGCTGACGGGTAAAT
Occludin	Forward primer: ACTATGCGGAAAGAGTTGACAG Reverse primer: GTCATCCACACTCAAGGTCAG
TNF-α	Forward primer: ACTCCAGGCGGTGCCTATG Reverse primer: GAGCGTGGTGGCCCCT
IL-6	Forward primer: CCAGTTGCCTTCTTGGGACT Reverse primer: GGTCTGTTGGGAGTGGTATCC
IL-1β	Forward primer: GTGGCTGTGGAGAAGCTGTG Reverse primer: GAAGGTCCACGGGAAAGACAC
CCL2	Forward primer: ACCTTTTCCACAACCACCT Reverse primer: GCATCACAGTCCGAGTCA
CCR2	Forward primer: AAGGGTCACAGGATTAGGAAG Reverse primer: ATGGTTCAGTCACGGCATA
RIP3	Forward primer: GAAGACACGGCACTCCTTGGTA Reverse primer: CTTGAGGCAGTAGTTCTTGGTGG
MLKL	Forward primer: CCTTGCTTGCTTGCTTTT Reverse primer: TTTCCTTGAGTTTGAGCCA

### Western Blotting

The liver and intestinal tissues were dissolved in RIPA, PMSF, and protease inhibitors. After homogenization, the protein concentrations were determined using bicinchoninic acid protein assay (Thermo Scientific Inc.). Proteins were separated using SDS-polyacrylamide gel electrophoresis system and then blotted onto a polyvinylidene fluoride membrane (Invitrogen, USA). Primary anti- RIP3(ab62344, Abcam, Cambridge, MA, USA), anti-MLKL (ab196436, Abcam, Cambridge, MA, USA), anti-TNF-α (ab183218, Abcam, Cambridge, MA, USA), anti-IL-6 (ab229381, Abcam, Cambridge, MA, USA), anti-ZO-1 (Abcam, USA), anti-Occludin (Abcam, USA), and anti-GAPDH (CST) antibody were then applied, and anti-GAPDH antibody was employed as the loading control. After incubation with horseradish peroxidase-conjugated secondary antibodies, the chemiluminescent signal was detected. Band intensity was determined by image processor program (Image J).

### Cell Isolation and Flow Cytometry Analysis

MAbs specific for CD45, CD11b, and F4/80 were obtained from BD Biosciences. Single-cell suspensions of lymphocyte were harvested from mouse liver. The cells were suspended in buffer, incubated with the above antibody for 30 min, and examined on a FACSCalibur flow cytometer (Becton Dickinson, USA). RAW264.7 cells seeded in a 12-well plate at a density of 1×10^5^ cells per well were stained with Annexin V/PI (BD Pharmingen, San Diego, CA, USA) to detect the apoptosis state in accordance with the manufacturer specifications. Data were analyzed using FlowJo 7.6 software.

### RIP3 siRNA Knockdown

Transient genetic silencing of RIP3 was performed by reverse transfection of RAW264.7 cells with 20–30 Nm Silencer^®^ Select siRNAs (Life Technologies, Inc.) using Lipofectamine^®^ RNAiMax reagent (Life Technologies, Inc.), and Opti-MEM^®^ medium (Life Technologies, Inc.). Negative nontarget siRNA was used as control. Knockdown efficiency was confirmed by Western blot analysis and qRT-PCR.

### Intestinal Microbiota Analysis

16S rRNA gene sequencing procedure was performed by GENEWIZ, lnc. (Suzhou, China). Total fecal bacteria DNA extractions were acquired by QIAamp ^®^ Fast DNA Stool Mini Kit (QIAamp, Germany). The microbial 16S V3-V4 region was amplified with indexes and adaptors-linked universal primers (341F: ACTCCTACGGGAGGCAGCA G,806R: GGACTACHVGGGTWTCTAAT). PCR was performed using KAPA HiFi Hotstart PCR kit high fidelity enzyme in triplicate. Amplicon libraries were quantified by Qubit 2.0 Fluorometer (Thermo Fisher Scientific, Waltham, US) and then sequenced on Illumina HiSeq platform (Illumina, San Diego, US) for paired end reads of 250 bp. After the singletons were discarded and the chimeras were removed, the tags were clustered into operational taxonomic units (OTUs) using USEARCH (v7.0.1090) at 97% similarity. A representative sequence of each OTU was subjected to taxonomy-based analysis using the RDP database. Heatmap was created using R. Cluster analysis. Alpha and beta diversities were analyzed using QIIME. The relative abundance of bacteria was expressed as the percentage.

### Statistical Analysis

Data were presented as mean ± SD. Comparisons among different groups were performed by unpaired one-way ANOVA or Student’s t-test using SPSS 22.0. p<0.05 was considered statistically significant.

## Results

### Increased Intestinal Permeability and Loss of Epithelial Barrier Integrity in AIH Patients

**Figure 1:**
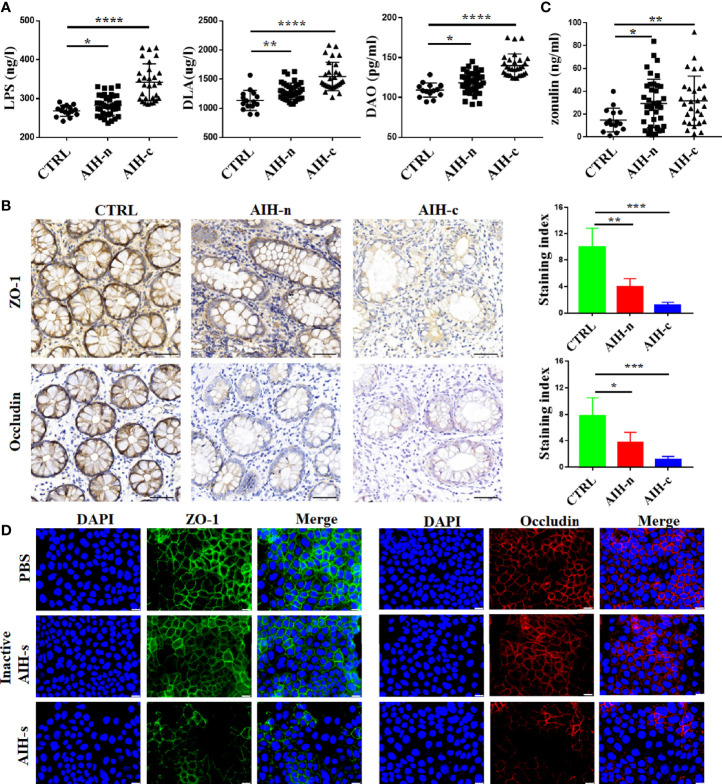
Increased intestinal permeability and loss of epithelial barrier integrity in AIH patients. **(A)** The plasma concentrations of LPS, DLA and DAO in CTRL group (n=15), AIH-n group (n=39) and AIH-c group (n=29). **(B)** Expression of ZO-1 and Occludin in the colon were assessed by immunostaining. **(C)** The plasma concentration of zonulin in the three groups. **(D)** Representative immunostaining of ZO-1 and Occludin in Caco-2 cells. Scale bars: 50μm. The data were presented as means ± SD (Student’s t-test, *p < 0.05, **p < 0.01, ***p < 0.001, ****p < 0.0001).

The main clinical and demographic features of enrolled patients and controls are shown in **Supplementary Table 1**. Plasma LPS, DLA, and DAO tests revealed an increased intestinal permeability in AIH-n group compared with that in the CTRL group (**Figure 1A**). To further assess the integrity of the intestinal barrier in these patients, we detected the structural proteins including ZO-1 and Occludin in the ileocecal junction through IHC staining (**Figure 1B**). The staining index of ZO-1 and Occludin in AIH-n group was significantly decreased compared with that in the CTRL group. Zonulin is the only physiological modulator of intercellular tight junctions involved in the trafficking of macromolecules and therefore in tolerance/immune response balance (27). Hence, the plasma zonulin levels were evaluated and was found to be significantly increased in the two AIH groups compared with that in the CTRL group (**Figure 1C**), indicating that breakage of the intestinal barrier integrity is an early event in the pathogenesis of AIH.

Previous data have demonstrated that treatment-naïve AIH patients had compositional and functional alterations of gut microbiome (28). To investigate the effects of gut dysbiosis on intestinal barrier function, we used a vitro model in which Caco-2 epithelial cell monolayers treated with the supernatant of feces from AIH patients (AIH-s). As shown in **Figure 1D**, the expressions of ZO-1 and Occludin were dramatically decreased in the AIH-s group compared to the control groups (inactive AIH-s group and PBS group).

### Activation and Infiltration of Macrophages in Liver Tissue of AIH Patients

**Figure 2:**
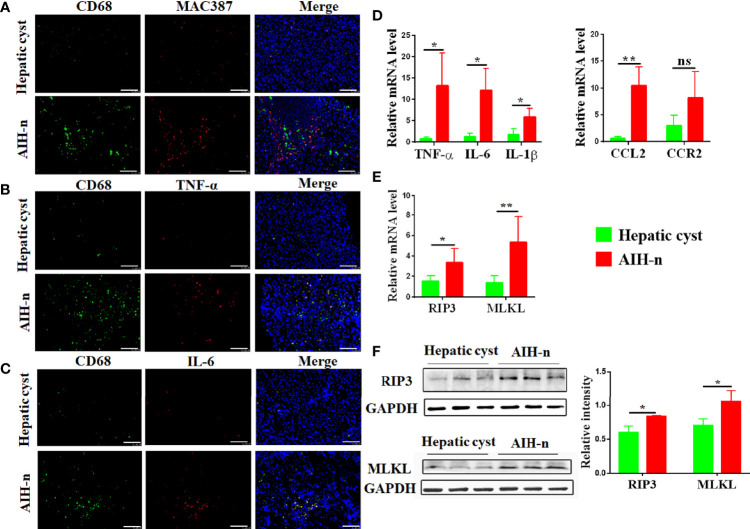
Activation and infiltrating of macrophages in the liver tissue of AIH patients. **(A–C)** Double-immunofluorescence staining for CD68 and MAC387 **(A)** CD68 and TNF-α **(B)** CD68 and IL-6 **(C)** in liver tissues of patients with AIH and hepatic cyst. **(D)** RT-qPCR analysis of TNF-α, IL-6, IL-1β, CCL2 and CCR2 on tissue homogenates from the liver of the two groups. **(E)** RT-qPCR analysis of RIP3 and MLKL in the liver of the two groups. **(F)** Protein levels of RIP3 and MLKL in the liver of the two groups were detected and the relative intensity was quantified. Scale bars: 100μm. The data were presented as means ± SD (Student’s t-test, *p < 0.05, **p < 0.01, ns: p < 0.05).

Liver macrophages, which consist of resident macrophages (Kupffer cells) and monocytes-derived macrophages, maintain liver immune homeostasis. Therefore, macrophage heterogeneity and activation status in the liver tissues of AIH patients were studied. Double immunofluorescence analyses for CD68 and MAC387 revealed that the number of resident and infiltrating macrophages significantly increased in the liver tissues of AIH-n group (**Figure 2A**). Furthermore, intracellular staining of inflammatory cytokines such as TNF-α and IL-6 co-stained with CD68 showed that the liver macrophages in AIH patients are significantly activated and a majority of the inflammatory signature derives from liver macrophages (**Figures 2B, C**). Besides, the mRNA expression of related inflammatory cytokines and chemokines in liver tissues was investigated (**Figure 2D**). TNF-α, IL-6, and IL-1β expression was significantly increased in the AIH-n group. CCL2 expression was also increased, and that of CCR2 was not significantly different.

RIP3 has been increasingly recognized as a key inflammatory signal adapter that mediates programmed necroptosis and the consequent release of inflammatory cytokines and chemokines (29, 30). Therefore, the activation of RIP3 and MLKL (the direct downstream effector of RIP3) in the liver tissues of AIH-n group was further explored. As shown in **Figure 2E**, the AIH-n group had significantly higher mRNA expression of RIP3 and MLKL than the hepatic cyst group. Concordantly, the protein expression of RIP3 and MLKL was also increased in the AIH-n group (**Figure 2F**).

### Breakage of Intestinal Barrier Augments Activation and Infiltration of Liver Macrophages

**Figure 3:**
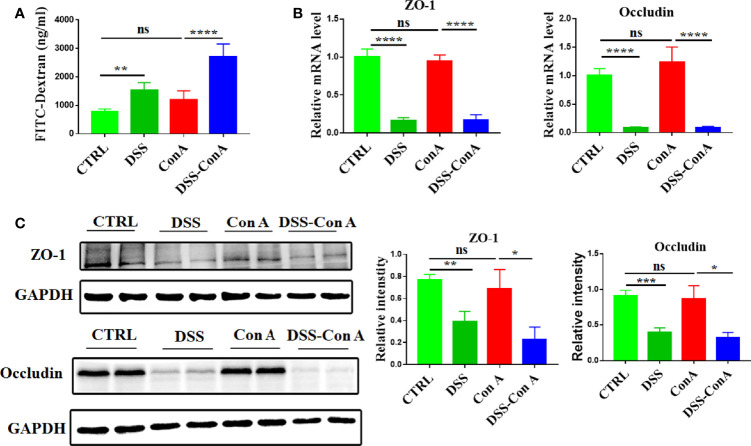
Breakage of intestinal barrier integrity by 1%DSS. **(A)** FITC-dextran *in vivo* permeability assay in the four groups. **(B)** RT-qPCR analysis of ZO-1 and Occludin on tissue homogenates from the colon of the four groups. **(C)** Protein levels of ZO-1 and Occludin in the colon of the four groups were detected and the relative intensity was quantified. (n=6). The data were presented as means ± SD (Student’s t-test, *p < 0.05, **p < 0.01, ***p < 0.001, ****p < 0.0001, ns: p < 0.05).

Clinical data indicated an increase in the intestinal permeability and alterations of liver immune homeostasis in AIH-n patients. Hence, we hypothesized that the enteropathy and breakage of the intestinal barrier integrity are not epiphenomena but could play a pathogenic role in AIH by regulating liver inflammation. For hypothesis testing, the tandem model of DSS-Con A was employed. As shown in **Supplementary Figure 1B**, the body weight of mice in each group did not differ significantly (p > 0.05). In addition, the liver index and spleen index were significantly increased in the Con A group compared with those in the CTRL group, but no difference was observed between the Con A and DSS-Con A groups (**Supplementary Figure 1C, D**). Furthermore, the gut barrier integrity of the tandem model was assessed. The results showed that the DSS-Con A group had significantly higher plasma concentration of FITC-dextran compared with the Con A group (**Figure 3A**). The mRNA and protein expression levels of ZO-1 and Occludin were significantly decreased in the gut mucosa of DSS-Con A group compared with those of Con A group (**Figures 3B, C**).

**Figure 4:**
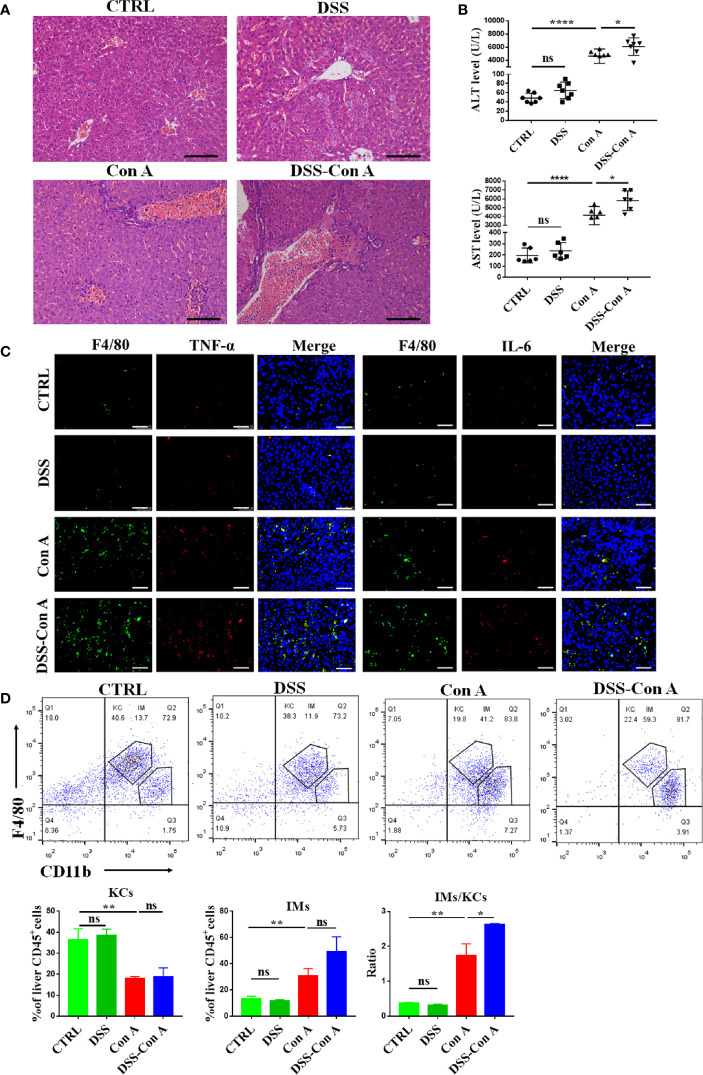
Breakage of the intestinal barrier aggravated liver injury and infiltration of liver macrophages. **(A)** HE staining of liver tissues in four groups. **(B)** The plasma concentrations of ALT and AST in four groups. **(C)** Double-immunofluorescence staining for F4/80 and TNF-α, F4/80 and IL-6 of liver tissues in four groups. **(D)** Representative flow cytometry plots and percentage of CD45^+^F4/80^hi^CD11b^lo^ Kupffer cells and CD45^+^F4/80^lo^CD11b^hi^ infiltrating macrophages in mononuclear cells from liver of the four groups. (n=6). Scale bars: 50μm. The data were presented as means ± SD (Student’s t-test, *p < 0.05, **p < 0.01, ****p < 0.0001, ns: p < 0.05).

Next, the inflammation and immunity of liver tissues were evaluated, and our data showed that the breakage of the intestinal barrier aggravated the Con A-mediated liver inflammation in the portal area (**Figure 4A**). The plasma transaminase levels of DSS-Con A group significantly increased compared with those of Con A group (**Figure 4B**). Moreover, the mice in the DSS-Con A group had a severe inflammatory cytokine milieu with higher mRNA expression of TNF-α, IL-6, and IL-1β in the liver tissues compared with that in Con A group, and the CCL2 expression was also significantly increased in DSS-Con A group (**Supplementary Figure 3A, B**). In addition, immunofluorescence staining of inflammatory cytokines in F4/80^+^ cells indicated that the liver macrophages in DSS-Con A group had a more severe inflammatory signature compared with that in Con A group (**Figure 4C**). The CD45^+^ F4/80^+^ CD11b^+^ population of liver mononuclear cells were analyzed *via* flow cytometry to examine the state of the resident and infiltrating macrophages in the liver following the intestinal barrier breakage. The number of CD45^+^ F4/80^hi^ CD11b^lo^ liver resident Kupffer cells was significantly decreased, whereas that of CD45^+^ F4/80^lo^ CD11b^hi^ infiltrating macrophages significantly increased in Con A group compared with those of CTRL group. However, no difference was found between Con A and DSS-Con A groups. The ratio of infiltrating macrophages to Kupffer cells significantly increased in DSS-Con A group compared with that in Con A group (**Figure 4D**). These results suggested that the intestinal barrier breakage contributes to Con A-mediated liver injury by promoting the activation and infiltration of liver macrophages.

### Intestinal Barrier Disruption Aggravates Activation of RIP3 Signaling Pathway of Liver Tissue

**Figure 5:**
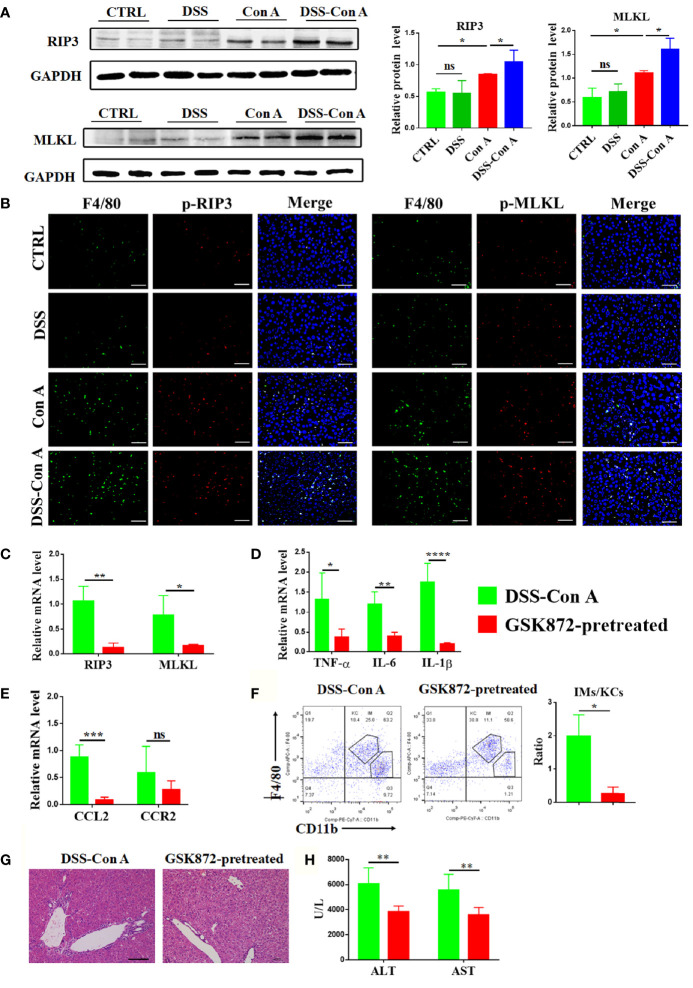
Breakage of intestinal barrier aggravated the activation of RIP3 signaling pathway of the liver tissue. **(A)** Protein levels of RIP3 and MLKL in the liver of the four groups were detected and the relative intensity was quantified. **(B)** Representative double-immunofluorescence staining for F4/80 and p-RIP3, F4/80 and p-MLKL of liver tissues in four groups. **(C)** RT-qPCR analysis of RIP3 and MLKL on liver tissue of DSS-ConA group and GSK872-pretreated group. **(D)** RT-qPCR analysis of TNF-α, IL-6, and IL-1β on liver tissue of the two groups. **(E)** RT-qPCR analysis of CCL2 and CCR2 on liver tissue of the two groups. **(F)** Representative flow cytometry plots and percentage of CD45^+^F4/80^hi^ CD11b^lo^ Kupffer cells and CD45^+^ F4/80^lo^ CD11b^hi^ infiltrating macrophages in mononuclear cells from liver of the two groups. **(G)** HE staining of the liver tissue from the two groups. **(H)** The plasma concentrations of ALT and AST of the two groups. (n=6). Scale bars: 50μm. The data were presented as means ± SD (Student’s t-test, *p < 0.05, **p < 0.01, ***p < 0.001, ****p < 0.0001, ns: p < 0.05).

The mechanism underlying the augmented activation and accumulation of liver macrophage was studied under this tandem model. The protein expression of RIP3 and MLKL in the liver tissue was significantly upregulated in DSS-Con A group compared with that in Con A group (**Figure 5A**). The relative mRNA expression of RIP3 and MLKL also increased (**Supplementary Figure 3C**). As shown in **Figure 5B**, p-RIP3-positive cells and p-MLKL-positive cells were markedly increased in the liver tissues from DSS-Con A group, particularly in macrophages. Further, the mice were treated with GSK872 to investigate whether the inhibition of RIP3 signaling pathway can ameliorate the activation and infiltration of liver macrophages in the DSS-Con A group. The liver of GSK872-pretreated group showed markedly diminished RIP3 and MLKL expression (**Figure 5C**) and significantly inhibited mRNA expression levels of inflammatory cytokines and chemokines including TNF-α, IL-6, IL-1β, and CCL2 (**Figures 5D, E**). GSK872 also inhibited the infiltration of CD45^+^ F4/80^lo^ CD11b^hi^ macrophages, leading to a significantly decreased ratio of infiltrating macrophages to Kupffer cells (**Figure 5F**). Accordingly, the GSK872-pretreated group had significantly reduced inflammation in the portal area of liver tissues and decreased plasma transaminases levels compared with the DSS-Con A group (**Figures 5G, H**). Besides, the annexin V/PI apoptosis assay for further assessment of cell death showed that breakage of the intestinal barrier significantly induced the late apoptosis of liver macrophages and GSK872 markedly decreased the early and late apoptosis rates (**Supplementary Figure 4**). All together, these results indicated that the pronounced activation and infiltration of liver macrophages in the DSS-Con A group are regulated by RIP3 signaling pathway.

### RIP3 Signaling Pathway Regulates the Expression of Macrophage-related Cytokines and Chemokines in RAW264.7 Cell Lines

**Figure 6:**
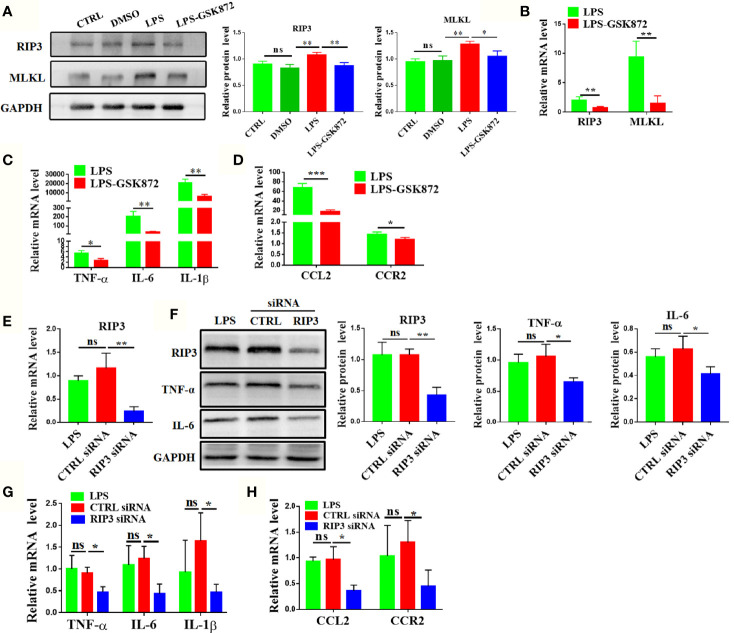
RIP3 signaling pathway regulates the expression of macrophage-related cytokines and chemokines in RAW264.7 cell lines. **(A, B)** Relative expression of RIP3 and MLKL was inhibited by GSK872. **(C, D)** Relative expression of TNF-α, IL-6, and IL-1β **(C)**, CCL2 and CCR2(D) was down-regulated by GSK872. **(E, F)** Relative expression of RIP3 was inhibited by RIP3 siRNA and key cytokines such as TNF-α and IL-6 were detected and the relative intensity was quantified. **(G, H)** Relative expression of TNF-α, IL-6, and IL-1β **(G)**, CCL2 and CCR2(H) was down-regulated by RIP3 siRNA. The data were presented as means ± SD of three independent experiments (Student’s t-test, *p < 0.05, **p < 0.01, ***p < 0.001, ns: p > 0.05).

The expression levels of macrophage-related cytokines and chemokines were analyzed by activating or inhibiting the RIP3 signaling pathway *in vitro* to further explore the effect of RIP3 signaling pathway on liver macrophages. The results showed that the relative mRNA expression of RIP3 and MLKL was upregulated after LPS stimulation in RAW264.7 cells (**Supplementary Figure 5A**). The protein level of RIP3 and MLKL also significantly increased in the LPS group (**Figure 6A**). Upon the activation of RIP3 signaling pathway by LPS, the relative mRNA expression levels of cytokines and chemokines including TNF-α, IL-6, IL-1β, CCL2, and CCR2 as well as the protein expression of key cytokines such as TNF-α and IL-6 were also significantly increased (**Supplementary Figure 5B, C**). By contrast, the relative mRNA expression of cytokines and chemokines were down-regulated when the RIP3 signaling pathway was inhibited with GSK872 (**Figures 6B–D**). The protein level of TNF-α and IL-6 in the LPS-GSK872 group also decreased but the difference was not statistically significant (**Supplementary Figure 5C**). Besides, the annexin V/PI apoptosis assay showed that LPS significantly induced the early and late apoptosis of RAW264.7 cells and GSK872 markedly decreased the late apoptosis rate (**Supplementary Figure 6**). Furthermore, RIP3 was efficiently knocked down by RIP3 siRNA when compared with CTRL siRNA (**Figures 6E, F**). Accordingly, the protein level of key cytokines such as TNF-α and IL-6 also significantly decreased in the RIP3 siRNA group (**Figure 6F**) and the relative mRNA expression of macrophage-related cytokines and chemokines including TNF-α, IL-6, IL-1β, CCL2, and CCR2 also significantly decreased in the RIP3 siRNA group (**Figures 6G, H**). All these results emphasized that the RIP3 signaling pathway can be activated by intestinal LPS to regulate the activation and accumulation of macrophages.

### RIP3-mediated Activation and Infiltration of Liver Macrophages Requires Gut Commensal Microbiota

**Figure 7:**
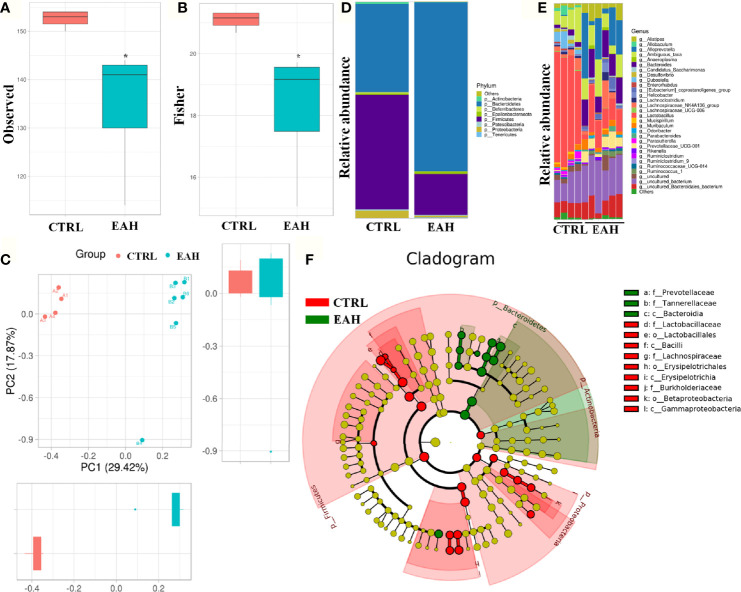
Alteration of the gut microbiota composition in EAH mice. **(A, B)** Observed **(A)** and Fisher **(B)** diversity indexes of the gut microbiota in CTRL group (n=4) and EAH group (n=6). **(C)** ANOSIM based on weighted UniFrac distances. **(D)** Bar charts of the gut microbiota composition at the phylum level in CTRL group and EAH group. **(E)** Bar charts of the gut microbiota composition at the genus level in each mouse. **(F)** Cladogram generated from the LEfSe. (*p < 0.05).

Disruption of the intestinal barrier lead to bacterial translocation, which consecutively activates immune cells to release various proinflammatory cytokines and chemokines (31). To test whether the gut commensal bacteria are required for RIP3 activation, EAH mouse model displaying dysbiosis in fecal microbiomes was used. **Figures 7A, B** shows that as measured by observed index and fisher index, the EAH group had significantly decreased alpha-diversity compared with the CTRL group. Principal component analysis based on weighted UniFrac distances revealed a different structure between the two groups (**Figure 7C**). The gut microbiota of all the samples in the two groups were dominated by three major phyla: Bacteroidetes, Firmicutes, and Proteobacteria (**Figure 7D**). Compared with those of the CTRL group, higher abundance of Bacteroidetes and lower abundance of Firmicutes and Proteobacteria were found in the EAH group. This phenomenon resulted in a decreased Firmicutes/Bacteroidetes (F/B) ratio (0.19) in the EAH group compared with that in the CTRL group (1.32). Genus-level analysis revealed that the mice in the EAH group had increased relative abundance of potential pathogenic bacteria, such as Bacteroides and Prevotellaceae_UCG-001, and a relatively low abundance of Lactobacillus and Ruminiclostridium (**Figure 7E**). Gut microbiota was also compared between the two groups through linear discriminant analysis effect size (LEfSe) to identify the specific microbiota linked to EAH. Prevotellaceae, which is associated with autoimmune diseases such as rheumatoid arthritis (32), were highly abundant in the EAH group (**Figure 7F**).

**Figure 8:**
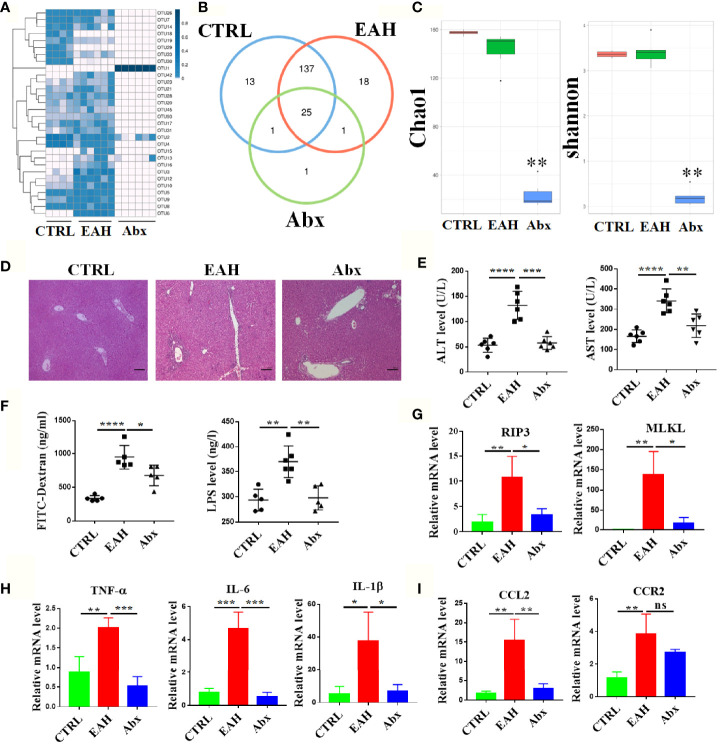
RIP3-mediated activation and infiltration of liver macrophages requires gut commensal microbiota. **(A, B)** Bacterial OUT heatmap **(A)** and Venn diagrams **(B)** in the CTRL group (n=4), EAH group (n=6) and Abx group (n=6). **(C)** The community richness and diversity of the gut microbiota were evaluated with Chao1 and Shannon indexes. **(D)** HE staining of the liver tissue from three groups. **(E)** The plasma concentrations of ALT and AST of three groups. **(F)**The plasma concentrations of FITC-D and LPS of three groups. **(G–I)** RT-qPCR analysis of RIP3 and MLKL **(G)** TNF-α, IL-6, and IL-1β **(H)** CCL2 and CCR2 **(I)** in three groups. Scale bars: 100μm. The data were presented as means ± SD (Student’s t-test, *p < 0.05, **p < 0.01, ***p < 0.001, ****p < 0.0001, ns: p > 0.05).

To analyze the role of gut commensal microbiota in liver immunity, the mice were treated with broad-spectrum antibiotic mixture prior to EAH induction. As shown in **Supplementary Figure 2B**, the mice in the EAH group had lower body weight than those in the CTRL group after 4 weeks, whereas no significant weight loss was found in the Abx group. In addition, the liver index and spleen index were significantly increased in the EAH group compared with those in the CTRL group, whereas no significant increase was observed in the Abx group (**Supplementary Figure 2C, D**). OTU comparison among the three groups revealed lower abundance of OTUs in the Abx group compared with those in the CTRL and EAH groups (28 OTUs vs. 176 OTUs vs. 181 OTUs). Among the 28 OTUs in the Abx group, 25 were shared by the three groups (**Figures 8A, B**). Analysis of Chao1 index and Shannon index indicated that the community richness and diversity also significantly decreased in the Abx group relative to those in the other two groups (**Figure 8C**). Remarkably, none of the mice in Abx group had developed hepatitis, and the transaminase level of the mice in the Abx group nearly returned to normal (**Figures 8D, E**). The intestinal permeability of these mice was also evaluated, and the results showed that the plasma FITC-D and LPS levels of Abx-treated mice were significantly decreased compared with those of the EAH mice (**Figure 8F**). The RIP3 signaling pathway was significantly inhibited with lower expression of inflammation cytokines and chemokines in liver tissues of the Abx group compared with those of the EAH group (**Figures 8G–I**). Besides, we evaluated the changes of liver macrophages by immunofluorescence and the results revealed that the mice in EAH group had increased number of macrophages in the liver tissue and the macrophages was significantly activated than that in the CTRL group, however, the broad-spectrum antibiotic mixture significantly alleviated the accumulation and activation of liver macrophages as shown in **Supplementary Figure 7**.

## Discussion

It’s known that alterations in the gut microbiota have been related with most autoimmune diseases, but in most cases, it remains unclear whether these changes are a cause or effect of the disease or merely a reflection of epidemiological differences between groups. The gut–liver axis has clinical importance as a potential therapeutic target in a wide range of chronic liver diseases (33–35). Recent evidence suggests that the intestinal environment specifically, modifications of the microbiome profile, regulate the pathogenesis of AIH by inducing intestinal inflammation and increasing gut permeability (36). In this present study, we try to explore the causality between the leaky gut/dysbiosis and AIH, demonstrating that loss of gut barrier integrity breaks liver immune homeostasis and augments liver injury.

The gut barrier is a fundamental gatekeeper to prevent translocation of bacterial components and the intestinal epithelial barrier (IEB) are crucial to prevent the passage of commensal bacteria and pathogens, from the lumen into the systemic circulation (37). The IEB is a single layer of epithelial cells held together by a complex junctional system composed of tight junctions, adherent junctions, and desmosomes (38). Recent studies indicated that leaky gut and increased intestinal permeability contributed to disease initiation and progression (39–41). Alterations of gut barrier integrity are found in patients affected by extraintestinal autoimmune diseases, but a direct causal link between enteropathy and triggering of autoimmunity is yet to be established. It was reported that, in mice models of T1D, loss of gut barrier integrity can lead to activation of islet-reactive T cells within the intestinal mucosa (42). Remarkably, Vieira et al. found that translocation of a gut pathobiont, Enterococcus gallinarum, to the liver and other systemic tissues triggers autoimmune responses in a genetic background predisposing to autoimmunity (43). In the present study, we demonstrated that the expression of tight junction proteins and the intestinal permeability is altered in AIH patients. Those alterations were detected at an early stage in AIH progression that is concomitant with the activation of the liver macrophages. Dysbiosis and reduction of tight junction proteins could ultimately lead to intestinal bacterial translocation with increased serum LPS levels and broken liver immune homeostasis that we observed in AIH patients and EAH mice.

The presence of loss of gut barrier integrity in patients and mice models of autoimmune diseases, such as T1D and systemic lupus erythematosus, has been known for long time but a causal link between the intestinal alterations and induction autoimmunity was never established (44). Our data demonstrate that loss of gut barrier integrity and modifications of the structural proteins prior to hepatitis, break liver immune tolerance, thus augment liver injury. Different triggering events have been reported such as viral infections or any factor that perturbs the liver environment leading to inflammation, tissue damage, and the release of sequestered liver antigen resulting in the stimulation of autoreactive immune cells (34). Here, we show that breakage of the gut barrier is one of those events that unleashes liver autoimmunity and provokes liver injury. The liver is particularly enriched in macrophages, which protect against infection, control host–microbiota mutualism, and maintain liver homeostasis (15, 45). Our data showed that intestinal barrier disruption increased activation and accumulation of liver macrophages thus aggravated liver injury. In the present study, we analyzed subsets of liver immune cells *via* flow cytometry and found the ratio of CD45^+^ F4/80^hi^ CD11b^lo^ infiltrating macrophages to CD45^+^ F4/80^lo^ CD11b^hi^ Kupffer cells significantly increased in DSS-Con A group.

RIP3 is an essential part of the cellular machinery that executes “programmed” or “regulated” necrosis. Bacterial products such as LPS can activate the RIP3 signaling pathway (46, 47). Upon activation, the necrosome complex phosphorylates MLKL, leading to fatal permeabilization of the plasma membrane that exerts pro-inflammatory functions (21). In agreement with the induced gene expression of necrosome-related genes, Western blot analyses of liver specimen revealed the elevated levels of RIP3 protein in DSS-Con A mice and EAH mice. Whether RIP3 regulates the activation and infiltration of liver macrophage in DSS-Con A mice remains unclear. Thus, these mice were treated with GSK872 to dampen the RIP3 activation and thus significantly ameliorated the activation and accumulations of liver macrophages. The same results were observed *in vitro*. Our data are in accordance with a recent report showing that the RIP3 expression is up-regulated in liver tissues and macrophages of humans and mice with liver fibrosis; in addition, the absence of RIP3 in macrophages could alleviate inflammation and macrophage or neutrophil accumulation in mice after carbon tetrachloride or bile duct ligation treatment (48). It’s worth noting that the link between the RIP3 pathway and AIH is not definitively clear without gene knock-out (KO). However, it’s reported that RIP3 regulates stem cells generation through modulating cell cycle progression genes and RIP3 KO displayed lower expression of cell cycle genes and a slower proliferation rate compared to wild type (49). Besides, Patrick-Simon Welz et al. demonstrated that genetic deficiency in RIP3 prevented the development of spontaneous pathology in both the small intestine and colon of mice, which may prevent us from studying the role of the intestinal barrier in AIH (50). For the above reasons, we used GSK872, a RIP3 specific inhibitor *in vivo* and *in vitro* experiments to illustrate the role of RIP3 pathway in AIH. Our data suggested that the loss of intestinal barrier augmented liver macrophage activation and infiltration and the RIP3 signaling pathway might be the underlying molecular mechanism of intestinal barrier disruption on AIH pathogenesis.

The gut microbiota has a strong impact in AIH pathogenesis as demonstrated both in humans and mice models, but it is still unclear how commensal bacteria modulate liver autoimmunity. In fact, while in other autoimmune diseases such as rheumatoid arthritis, autoimmune encephalomyelitis and multiple sclerosis, the gut microbiota plays a clear triggering role (51, 52). In AIH, key epitopes that might trigger the disease might be sought among environmental agents especially within the intestinal microbiome (1, 53). Our data showed that gut inflammation by itself in EAH mice depleted of endogenous microbiota is not capable to activate liver autoimmunity, which suggests that the commensal gut microbiota is required for the activation of innate immune response. The intestinal microbiota maintains gut barrier integrity, shapes the mucosal immune system and balances host defense with microbial metabolites, components, and attachment to host cells (54). While the role of gut microbiota in liver immunity is still controversial. In some cases, it plays a beneficial effect, for example, in SPF and gnotobiotic mice, gut microbiota and commensal D-lactate programs Kupffer cells to capture and kill circulating pathogens (55). On the contrary, the gut microbiota contributes to a mouse model of spontaneous bile duct inflammation and GF mice develop a milder biliary affection, thus suggesting that commensal strains are important to trigger liver autoimmunity (56).

Our study showed that the intestinal barrier and gut microbiota in patients with AIH and murine models were destroyed. Diminished intestinal barrier function contributed to the activation and accumulation of liver macrophages *via* RIP3 signaling pathway. This phenomenon further aggravated the immune response in the inflamed liver. Our results revealed the novel mechanism of immune tolerance breakage in the liver *via* the gut–liver axis. In addition, therapeutic and research potentials of regulating the intestinal microbiota for AIH therapy were explored.

## Data Availability Statement

We have uploaded the 16S rRNA data into the SRA database successfully. The SRA records will be accessible with the following link: https://www.ncbi.nlm.nih.gov/sra/PRJNA678135.

## Ethics Statement

The studies involving human participants were reviewed and approved by the Animal Ethical and Welfare Committee of Tianjin Medical University. The patients/participants provided their written informed consent to participate in this study. The animal study was reviewed and approved by the Animal Ethical and Welfare Committee of Tianjin Medical University.

## Author Contributions

LZ and BW designed the study. HZ, ML, WZ and YZ performed the experiments. YL, LG, YZ, YR, WC and JZ analyzed the results. HZ and LZ wrote the paper. All authors contributed to the article and approved the submitted version.

## Funding

This study was supported by the grants (81860109 and 81470834) from the National Natural Science Foundation of China.

## Conflict of Interest

The authors declare that the research was conducted in the absence of any commercial or financial relationships that could be construed as a potential conflict of interest.
