# Supplementary material for: Leaky Gut Driven by Dysbiosis Augments Activation and Accumulation of Liver Macrophages via RIP3 Signaling Pathway in Autoimmune Hepatitis
Source: Front Immunol. 2021 Mar 25;12:624360. doi: 10.3389/fimmu.2021.624360 (PMC8027109; doi:10.3389/fimmu.2021.624360)
Supplement: Supplementary file 1 [file Image_1.pdf]

Supplementary Material

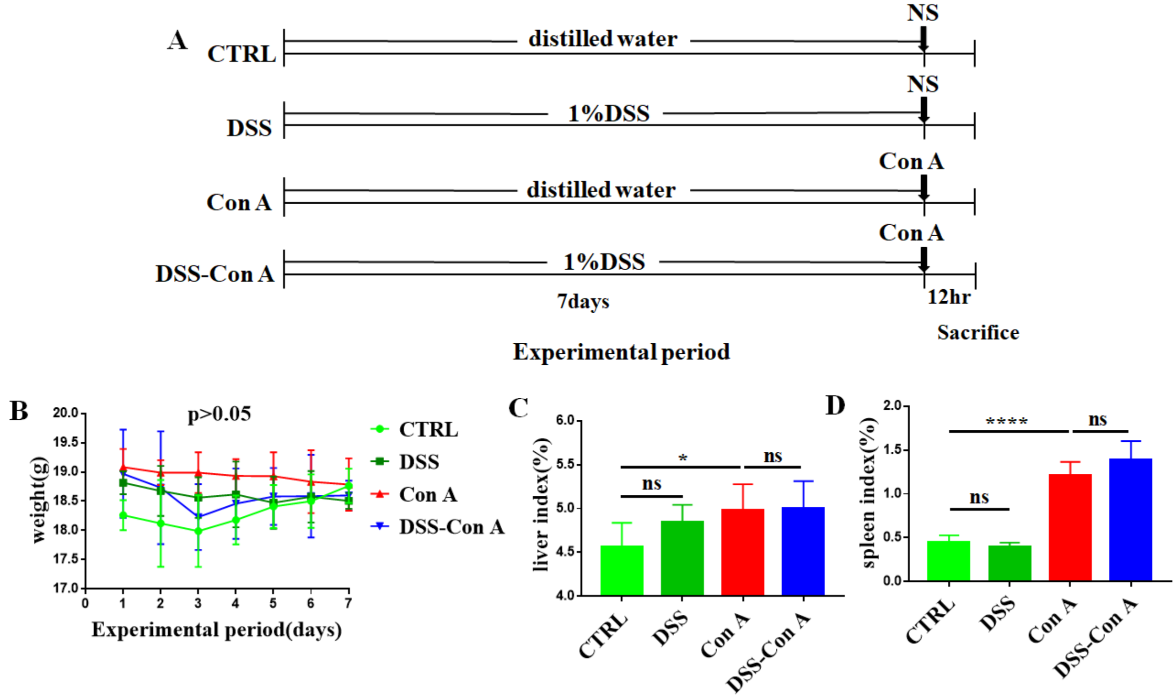

Supplementary Figure 1. The experimental process of DSS-Con A model and general state of mice. (A) The modeling process of DSS-Con A. (B) Body weight of each group was recorded daily. (C,D) Comparison of liver index(C) and spleen index(D) between groups. (n=6). The data were presented as means ± SD (Student's t-test, * $p<0.05$, **** $p<0.0001$).

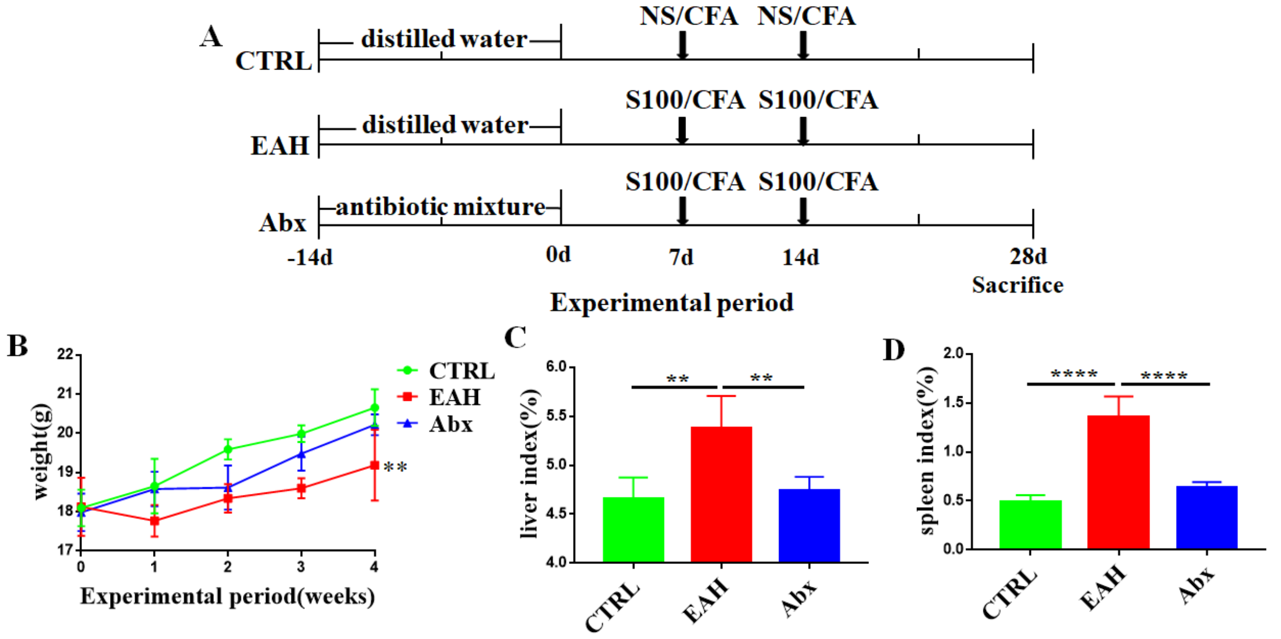

Supplementary Figure 2. The experimental process of EAH model and general state of mice. (A) Pre-administration of antibiotics mixture for two weeks and modeling process of EAH during 4 weeks. (B) Body weight of each group was recorded weekly. (C,D) Comparison of liver index(C) and spleen index(D) between groups. (n=6). The data were presented as means ± SD (Student's t-test, ** $p<0.01$, **** $p<0.0001$).

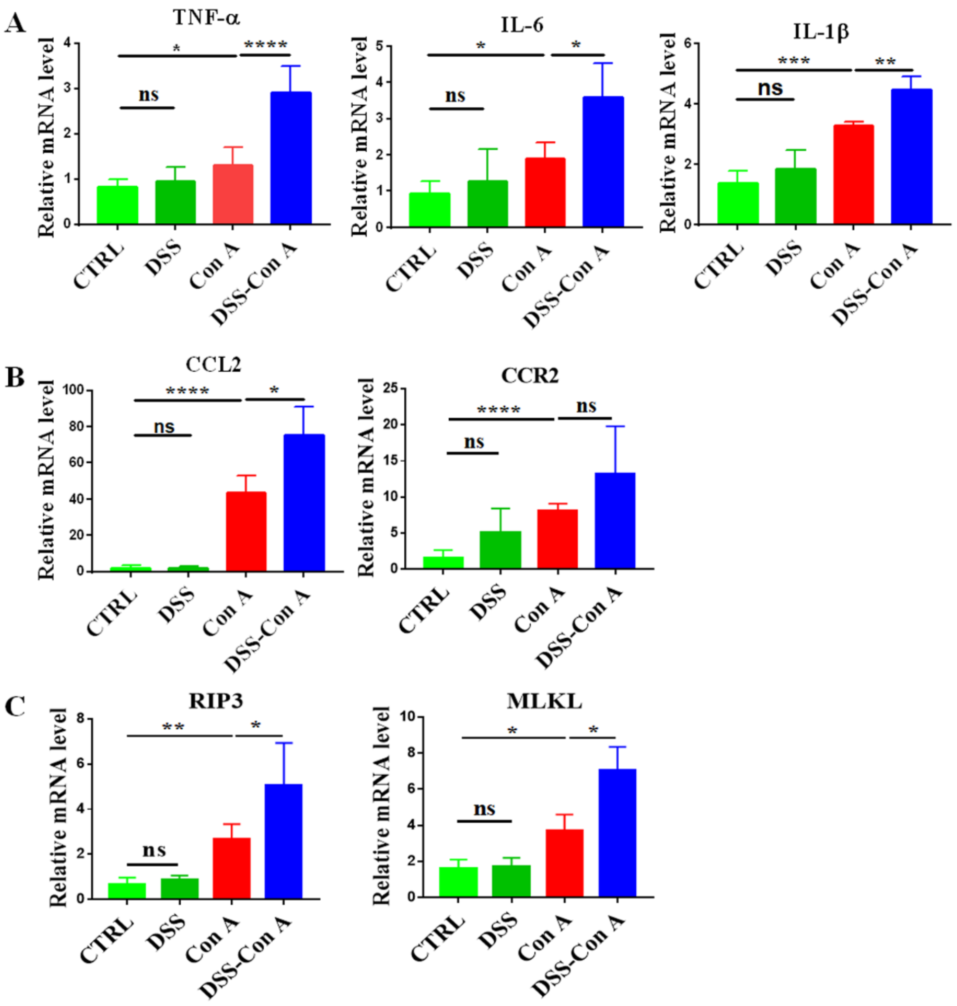

Supplementary Figure 3. (A-C) RT-qPCR analysis of TNF-α, IL-6, and IL-1β(A), CCL2 and CCR2(B), RIP3 and MLKL(C) on tissue homogenates from the liver of the four groups. (n=6). The data were presented as means ± SD (Student's t-test, * $p<0.05$, ** $p<0.01$, *** $p<0.001$, **** $p<0.0001$).

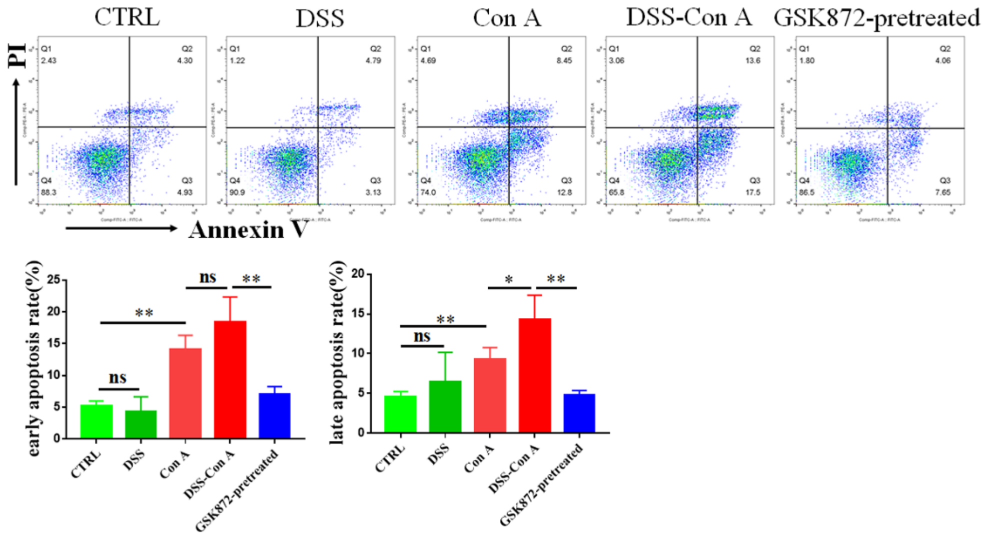

Supplementary Figure 4. Apoptosis of liver macrophages in the five groups was detected with Annexin V/PI and FACS. Breakage of the intestinal barrier significantly induced the late apoptosis of liver macrophages and GSK872 markedly decreased the early and late apoptosis rate. (n=6). The data were presented as means ± SD (Student's t-test, * $p<0.05$, ** $p<0.01$).

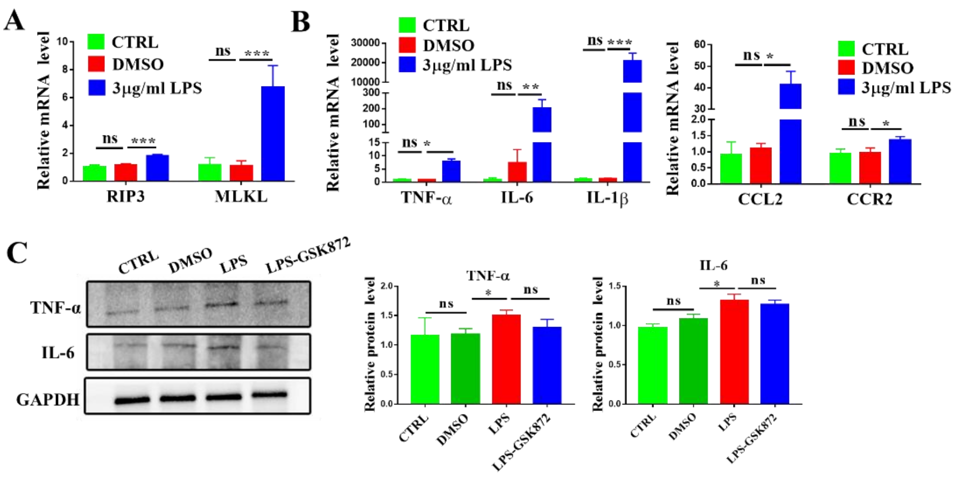

Supplementary Figure 5. RIP3 signaling pathway regulates the expression of macrophage-related cytokines and chemokines in RAW264.7 cell lines. (A, B) RT-qPCR analysis of RIP3 and MLKL(A), TNF-α, IL-6, IL-1β, CCL2 and CCR2(B) in RAW264.7 cells of CTRL group, DMSO group and LPS group. (C) The protein level of RIP3, MLKL and key cytokines such as TNF-α and IL-6 were detected and the relative intensity was quantified. The data were presented as means ± SD of three independent experiments (Student’s t-test, * $p<0.05$, ** $p<0.01$, *** $p<0.001$).

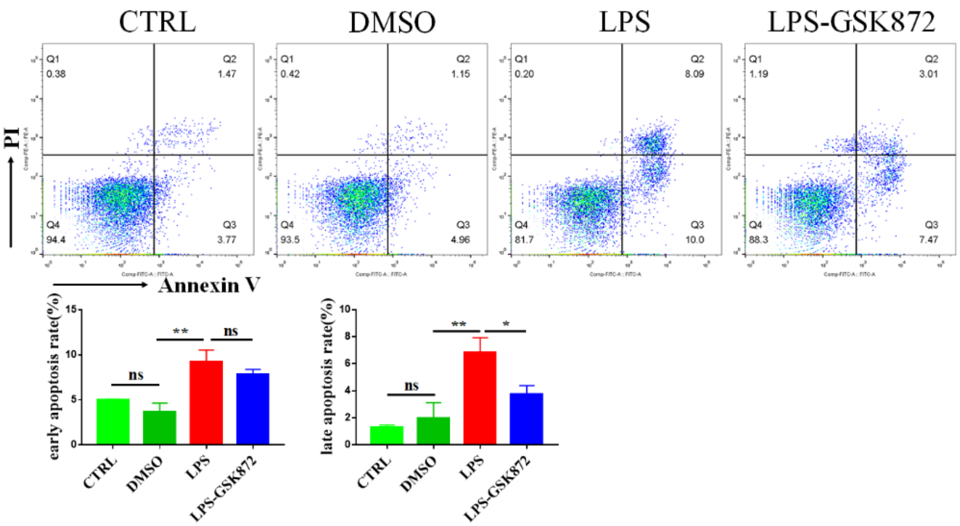

Supplementary Figure 6. Apoptosis of RAW264.7 cells was detected with Annexin V/PI and FACS. LPS significantly induced the early and late apoptosis of RAW264.7 cells and GSK872 markedly decreased the late apoptosis rate. The data were presented as means ± SD of three independent experiments (Student's t-test, * $p<0.05$, ** $p<0.01$).

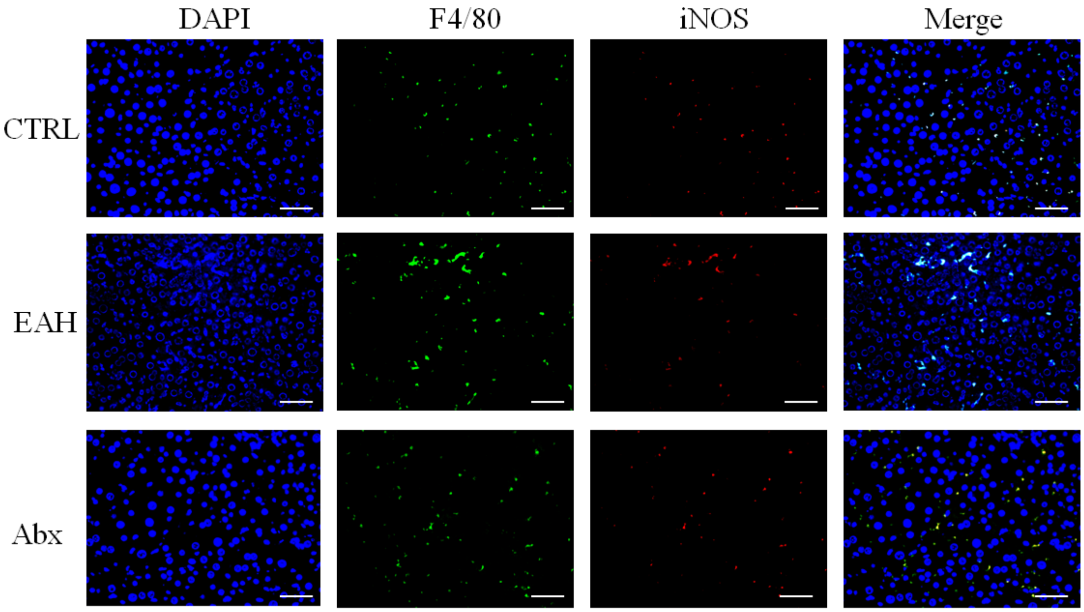

Supplementary Figure 7. Activation and accumulation of macrophages in the liver tissue of mice among the three groups via double-immunofluorescence staining for F4/80 and iNOS. (n=6). Scale bars: 100µm.

Supplementary Table 1

Characteristics of the participants enrolled

	CTRL（n=15）	AIH-n（n=39）	AIH-c（n=29）	*P* value
Age（year）	60.3±8.1	58.9±8.4	62.4±7.1	0.194
Sex(male/female)	0/15	2/37	4/25	0.193
WBC($\times 10^9$/L)	5.0±0.3	5.3±1.1	4.1±1.5	0.001
Hgb (g/L)	128.4±8.0	130.1±15.5	111.6±24.5	0.000
PLT ($\times 10^9$/L)	220.1±45.6	210.6±72.4	110.1±55.9	0.000
ALB(U/L)	40.3±3.0	40.3±5.1	33.5±9.1	0.000
GLO (U/L)	35.8±5.1	34.4±7.5	36.7±5.8	0.378
ALT (U/L)	14(8~16)	32.5(20.8~103.2)	23(18~38)	0.000
AST (U/L)	17(15~20)	35(24~69.8)	34(26~68)	0.000
ALP (U/L)	71.8±17.4	83.4±24.8	77.9±28.7	0.297
GGT (U/L)	31.3±14.2	35.6±20.5	26.0±12.9	0.095
TBIL(mg/dl)	13.2(10.8~15.4)	13.6(10.2~26)	16.3(12~26.2)	0.107
IgG (mg/dl)	NA	1785.7±683.0	2041.3±635.1	0.148
anti-ANA (%)	NA	90(35/39)	100(29)	0.130
anti-SMA (%)	NA	15(5/33)	8.7(2/23)	0.688

WBC: White blood cells; Hgb: Hemoglobin; PLT: Platelets; ALB: Albumin; GLO: globulin; ALT: Alanine transaminase; AST: Aspertate aminotransferase; ALP: Alkaline phosphatase; GGT: amma-glutamyl transpeptidase; TBIL: total bilirubin; IgG: immunoglobulin G; anti-ANA: Anti-nuclear antibodies; anti-SMA: Anti-smooth muscle antibody.
